# Diesel exhaust particulate increases the size and complexity of lesions in atherosclerotic mice

**DOI:** 10.1186/1743-8977-10-61

**Published:** 2013-12-11

**Authors:** Mark R Miller, Steven G McLean, Rodger Duffin, Akeem O Lawal, Jesus A Araujo, Catherine A Shaw, Nicholas L Mills, Ken Donaldson, David E Newby, Patrick WF Hadoke

**Affiliations:** 1 Centre for Cardiovascular Sciences, University of Edinburgh, 47 Little France Crescent, EH16 4TJ Edinburgh, Scotland, UK; 2 Centre for Inflammation Research, University of Edinburgh, Edinburgh, Scotland, UK; 3 Division of Cardiology, David Geffen School of Medicine at UCLA, Los Angeles, CA, USA

**Keywords:** Diesel, Air pollution, P, Atherosclerosis, ApoE, Oxidative stress

## Abstract

**Objective:**

Diesel exhaust particulate (DEP), a major component of urban air pollution, has been linked to atherogenesis and precipitation of myocardial infarction. We hypothesized that DEP exposure would increase and destabilise atherosclerotic lesions in apolipoprotein E deficient (ApoE^−/−^) mice.

**Methods:**

ApoE^−/−^ mice were fed a ‘Western diet’ (8 weeks) to induce ‘complex’ atherosclerotic plaques, with parallel experiments in normal chow fed wild-type mice. During the last 4 weeks of feeding, mice received twice weekly instillation (oropharyngeal aspiration) of 35 μL DEP (1 mg/mL, SRM-2975) or vehicle (saline). Atherosclerotic burden was assessed by en-face staining of the thoracic aorta and histological examination of the brachiocephalic artery.

**Results:**

Brachiocephalic atherosclerotic plaques were larger in ApoE^−/−^ mice treated with DEP (59±10%) than in controls (32±7%; *P* = 0.017). In addition, DEP-treated mice had more plaques per section of artery (2.4±0.2 *vs* 1.8±0.2; *P* = 0.048) and buried fibrous layers (1.2±0.2 *vs* 0.4±0.1; *P* = 0.028). These changes were associated with lung inflammation and increased antioxidant gene expression in the liver, but not with changes in endothelial function, plasma lipids or systemic inflammation.

**Conclusions:**

Increased atherosclerosis is caused by the particulate component of diesel exhaust producing advanced plaques with a potentially more vulnerable phenotype. These results are consistent with the suggestion that removal of the particulate component would reduce the adverse cardiovascular effects of diesel exhaust.

## Background

Particulate matter (PM) air pollution is associated with an increase in cardiovascular morbidity and mortality [1,2]. In epidemiologic studies, residents of areas with high air pollution have evidence of increased atherosclerosis [3-6]. This relationship is supported by pre-clinical studies showing that exposure to traffic-derived pollutants can increase atherosclerotic lesion size and alter lesion composition (comprehensively reviewed in [7]). The mechanisms responsible for these changes remain unclear, but there is evidence implicating altered endothelial cell function [8] and increased oxidative stress [9-11].

Diesel exhaust (DE) emissions are the major source of nanoparticles in the urban environment that, while contributing little to the airborne mass, produce very high numbers of ambient particles and a large reactive surface area, as well as the potential to deposit throughout the lungs [12,13]. We have previously shown that inhalation of environmentally-relevant concentrations of DE impairs endothelial and fibrinolytic function [14,15], promotes blood thrombogenicity [16] and exacerbates cardiac ischemia [17] in man. Inhaled DE also promotes atherosclerosis in mice [10,18-20]. Importantly, the procoagulant changes and impairment of vascular function induced by DE can be prevented by filtration [21,22], consistent with the hypothesis that the particulate component of DE drives the adverse cardiovascular effects (reviewed in [23]). To date, no investigation has directly determined the effect of diesel exhaust particulate (DEP) on the development of atherosclerotic lesions. We, therefore, addressed the hypothesis that exposure to DEP would increase lesion size and complexity in a murine model of atherosclerosis, and that this would be mediated by inflammation, endothelial dysfunction and oxidative stress.

## Results

### Instillation and pulmonary inflammation

**Figure 1:**
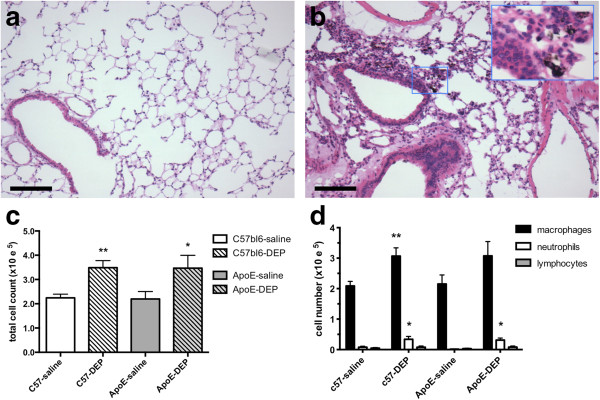
**Instillation of DEP induces pulmonary inflammation and alveolar remodelling.** Representative lung sections from **(a)** saline-instilled and **(b)** DEP-instilled ApoE^−/−^ mice showing airway fibrosis and cuffing of pulmonary blood vessels. Inset: High power image showing particle-laden macrophages deep within the airspaces. Scale bars = 100 μm. **(c)** Total cell counts in BALF. **(d)** Cell differentials in BALF. Mean ± S.E.M. (n = 7-9 for all) *P < 0.05, **P < 0.01, unpaired t- test comparing DEP with saline control in same animal type. ApoE^−/−^ were fed Western diet for 8 weeks, whereas wild-type mice were fed normal chow.

Pulmonary instillation of DEP was chosen as the exposure method; to administer only the particulate components of diesel exhaust, to ensure the complete delivery of the dose to the lungs, and establish this model as a basis for future experiments looking at the effects of fractionated DEP. Instillation was well tolerated, with no mortality and no effect on body weight (Additional file 1: Figure S1). Lungs of DEP-instilled mice showed fibrotic thickening of the alveolar septae and cuffing of alveolar blood vessels (Figure 1a,b). Particulate was visible deep in the airways, largely within alveolar macrophages. Instillation of DEP produced similar increases in the total cell count in bronchoalveolar lavage fluid (BALF) from atherosclerosis-prone apoliprotein E knockout (ApoE^−/−^) mice to that from wild-type (C57bl6) mice (*P* < 0.03, unpaired *t*-test; n = 7-9; Figure 1c). Macrophages were the predominant cell type in BALF (Figure 1d), although DEP also increased the number of neutrophils (*P* < 0.011) but not lymphocytes (*P* > 0.23) in both strains (n = 7-9 for all). DEP had no effect on interleukin-6 (IL-6; *P* > 0.13; n = 4-6 for all), tumour necrosis factor alpha (TNFα; *P* > 0.25; n = 4-5 for C57bl6, n = 6-7 for ApoE^−/−^) or monocyte chemotactic protein-1 (MCP-1; CCL2/JE; P > 0.23; n = 4-5 for all) levels in BALF (Additional file 1: Table S2).

### Distribution and size of atherosclerotic plaques

**Figure 2:**
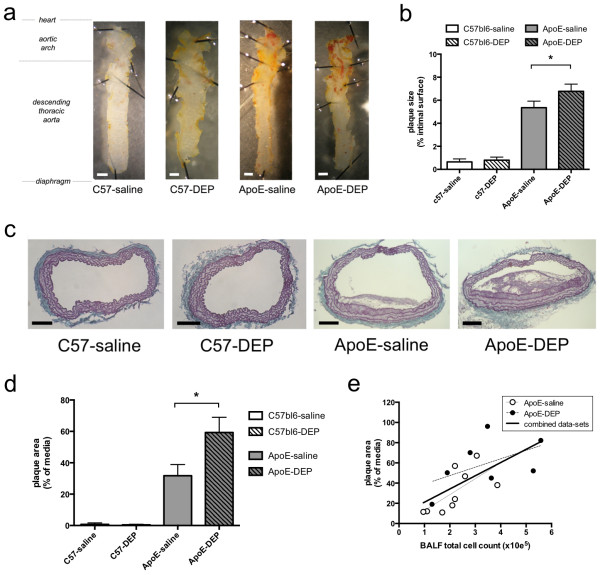
**Instillation of DEP increases atherosclerosis. (a)** Representative images showing lipid-rich lesions in the aorta following staining with Sudan IV (red stain; orange is non-specific staining). Scale bars = 1 mm. **(b)** Percentage of the intimal surface staining positive for lipids. Mean ± S.E.M. (n = 3-4 for C57bl6, n = 6-7 for ApoE^−/−^), ^*^P < 0.05, Mann–Whitney test, saline vs DEP in ApoE^−/−^ mice. **(c)** Representative transverse sections of atherosclerotic plaques in the brachiocephalic artery stained with United States Trichrome (UST). Scale bars = 100 μm. **(d)** Plaque size, as a percentage of medial area was increased in DEP-treated ApoE^−/−^ mice. Mean ± S.E.M. (n = 3-5 for C57bl6, n = 7-9 for ApoE^−/−^), ^*^P < 0.05, unpaired t-test, saline vs DEP in ApoE^−/−^ mice. Mean value obtained from analysis of serial sections at 100 μm intervals for each animal; Mean number of sections per group = 4.4 ± 0.6 for ApoE-saline (n = 9), 4.4 ± 0.8 for ApoE-DEP (n = 7). ApoE^−/−^ were fed Western diet for 8 weeks, whereas wild-type mice were fed normal chow. **(e)** Plaque size in the brachiocephalic artery correlated with the extent of pulmonary inflammation (BALF total cell count) at time of necropsy (R^2^ = 0.45, *P* = 0.0043, n = 16).

Aortae from C57bl6 mice exhibited little Sudan IV staining in either the saline- or DEP-instilled groups. In saline-treated ApoE^−/−^ mice, lipid-rich lesions were visible on the surface of the thoracic aorta largely confined to the aortic arch (Figure 2a). Lipid incorporation was greater in DEP- than saline-instilled ApoE^−/−^ mice (*P* = 0.046, n = 6-7; Figure 2b).

The brachiocephalic artery, which develops complex atherosclerotic plaques, was chosen for histological analysis [24]. No lesions were evident in C57/bl6 mice (saline, n = 3; DEP, n = 5). In contrast, ApoE^−/−^ mice developed large lesions throughout the length of the brachiocephalic artery, particularly in the 400 μm proximal to the bifurcation of the aortic arch (Figure 2c). Mean plaque size was markedly increased (*P* = 0.017) in DEP-treated ApoE^−/−^ mice (59±10%, n = 7) compared with controls (32±7%, n = 9; Figure 2d). This increase was seen using several different measures of lesion size (Additional file 1: Table S3). The size of atherosclerotic lesions was associated with levels of pulmonary inflammation (BALF total cell count) on the day of necropsy (*P* = 0.004, n = 16; Figure 2e).

### Plaque composition and structure

**Figure 3:**
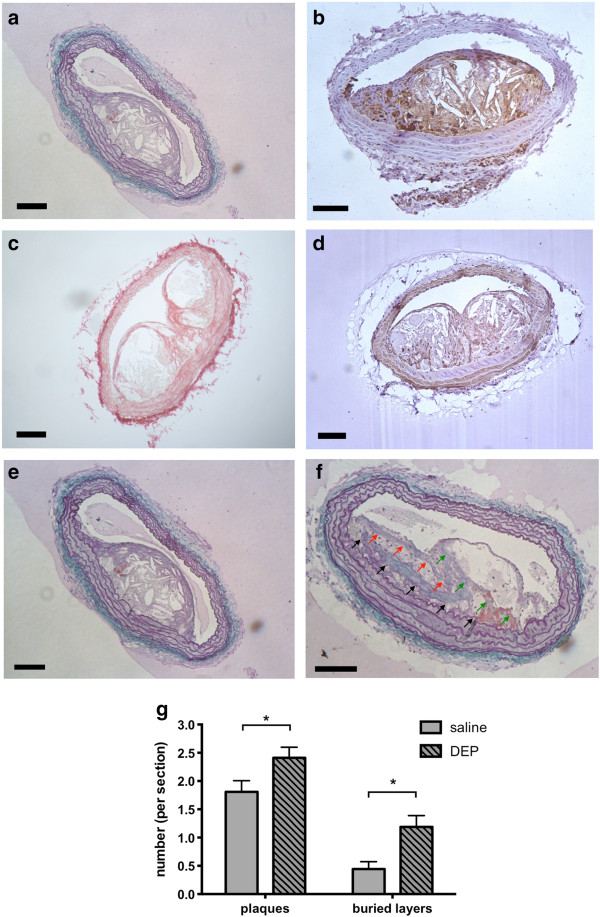
**Assessment of plaque composition and structure.** Representative brachiocephalic plaque sections showing **(a)** lipid cavities: plaque regions showing absence of all components of UST stain, **(b)** inflammatory cells: MAC2 immunohistochemistry (brown), **(c)** collagen: picrosirius red (red stain), **(d)** smooth muscle: SMA immunohistochemistry (brown). Scale bar = 100 μm. **(e)** Example of a large atherosclerotic plaque from a saline-treated ApoE^−/−^ mouse, showing only a single plaque per section and no buried fibrous layers. **(f)** Example of three buried fibrous layers in a plaque (black, green and red arrows mark each fibrous layer) taken from a DEP-treated ApoE^−/−^ mouse. UST staining, scale bar = 100 μm. **(g)** DEP instillation increased the average number of distinct plaques within each cross-section of lesion and the number of buried fibrous layers per section. Mean ± S.E.M. Values obtained from analysis of serial sections at 100 μm intervals for each animal; Mean number of sections per group = 4.4 ± 0.7 for ApoE-saline (n = 8), 4.4 ± 0.8 for ApoE-DEP (n = 7), *P < 0.05, Mann–Whitney test comparing saline with DEP. All images shown are from arteries from DEP-instilled ApoE^−/−^ mice, except panel 3e, taken from saline-treated ApoE^−/−^ mouse.

Plaque composition was studied in detail to provide insight into the potential ability of DEP to promote plaque instability. ApoE^−/−^ mice developed complex atherosclerotic lesions with variable proportions of smooth muscle cells, foam cells, lipid, cholesterol crystals and fibrous tissue (Figure 3). Exposure to DEP had little effect on lesion composition (n = 5-6/group; Additional file 1: Figure S2). Quantification of foam cells (MAC-2-positive), lipid cavities, smooth muscle cells and collagen (Figure 3a-d) showed no differences between saline- and DEP-treated ApoE^−/−^ mice (n = 7-9/group; Additional file 1: Figure S2). Similarly, no differences were detected in the levels of matrix metalloproteinases (MMP-2 and MMP-9; saline n = 9, DEP n = 4; Additional file 1: Figure S3) or fibrin/ fibrinogen (Additional file 1: Figure S4) in the plaques of DEP-treated mice (n = 5-6/group for all). However, DEP-treated animals had a greater number of distinct plaques per section of lesion (2.4±0.2 plaques) compared with saline-treated controls (1.8±0.2 plaques; *P* = 0.022, n = 7-8, Figure 3e-g). DEP also trebled the frequency of buried fibrous layers per section of lesion (1.2±0.2 vs 0.4±0.1; *P* = 0.013, n = 7-8; Figure 3e-g).

### Blood lipids, C-reactive protein and fibrinogen

**Figure 4:**
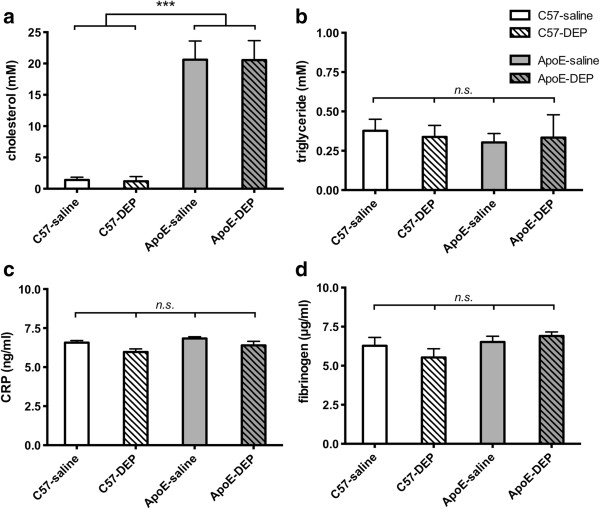
**DEP instillation did not alter lipid or markers of systemic inflammation and coagulation within the blood. (a)** Serum cholesterol (n = 10), **(b)** serum triglyceride (n = 10), **(c)** serum CRP (n = 8) and **(d)** plasma fibrinogen (n = 8) levels. Mean ± S.E.M. ***P < 0.001, unpaired t-test of C57bl6 vs ApoE; ^ns^*P* > 0.05, Bonferroni post-hoc tests, following one-way ANOVA. ApoE^−/−^ were fed Western diet for 8 weeks, whereas wild-type mice were fed normal chow.

Serum cholesterol concentrations were increased in ApoE^−/−^ mice fed a Western diet (20.1±0.7 mM) compared to C57bl6 mice fed standard chow (1.3±1.5 mM; n = 16, combined saline and DEP, for both genotypes; P < 0.001, Figure 4a) whereas serum triglyceride concentrations were similar in both ApoE^−/−^ (0.34±0.03 mM) and C57bl6 mice (0.36±0.02 mM; n = 16, combined saline and DEP, for both genotypes; Figure 4b). Both variables were unaffected by DEP instillation. Similarly, serum C-reactive protein (CRP) and plasma fibrinogen concentrations were unaffected by DEP instillation (both *P* > 0.05; n = 8 for all; Figure 4c,d; Additional file 1: Table S2). Total and active tissue plasminogen activator (t-PA) levels were below the limit of detection (n = 7-8 for all; Additional file 1: Table S2).

### Vascular function

**Figure 5:**
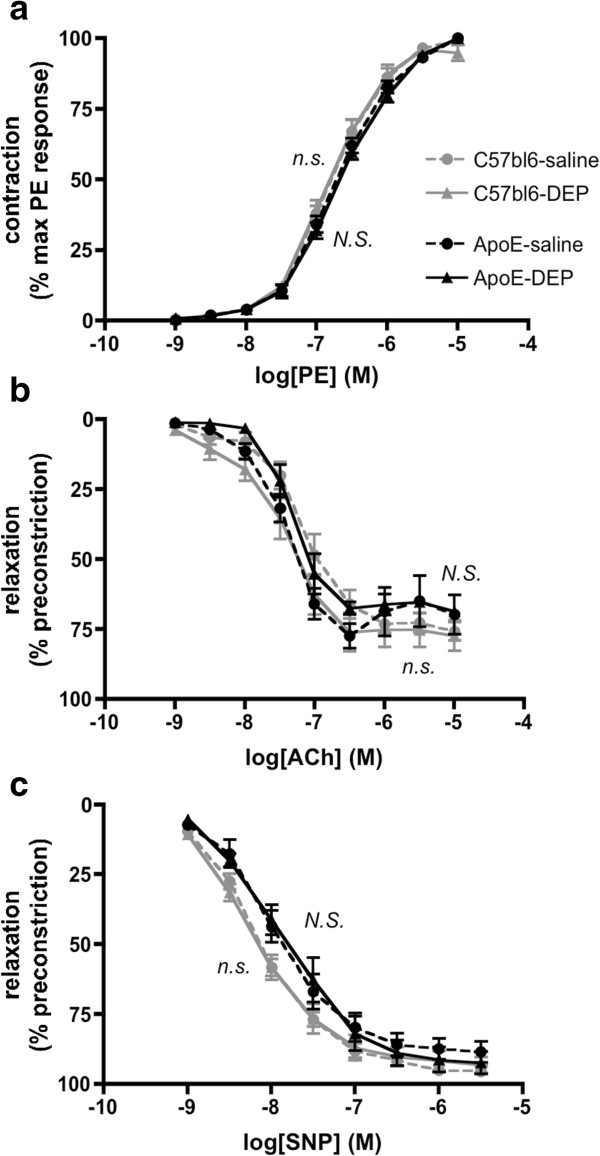
**Vascular function was unchanged by DEP instillation. (a)** Contraction of aortic rings to phenylephrine, **(b)** endothelium-dependent relaxation to acetylcholine and **(c)** endothelium-independent relaxation to sodium nitroprusside. Responses in C57bl6 mice (grey lines) or ApoE^−/−^ mice (black lines) receiving either saline (circles, broken line) or DEP (triangles, solid line) instillation. All dose response curves were performed in the same vessel from a single animal. ApoE^−/−^ were fed Western diet for 8 weeks, whereas wild-type mice were fed normal chow. Mean ± S.E.M. (n = 6-7 for phenylephrine, n = 4-5 for acetylcholine and SNP), ^n.s.^*P* > 0.05, two-way ANOVA saline vs DEP in C57bl6 mice, ^N.S.^*P* > 0.05, two-way ANOVA saline vs DEP in ApoE^−/−^ mice.

To determine whether DEP-instillation caused endothelial dysfunction, aortic rings were isolated for functional analysis. Phenylephrine caused an identical concentration-dependent contraction in C57bl6 and ApoE^−/−^ mice and was unaltered by DEP instillation (*P* > 0.24, DEP vs saline; n = 6-7; Figure 5a). Relaxations to the endothelium-dependent vasodilator, acetylcholine, or the endothelium-independent nitric oxide (NO) donor, sodium nitroprusside (SNP), were unaffected by DEP-instillation in C57bl6 and ApoE^−/−^ mice (*P* > 0.29; n = 4-5 for all; Figure 5b,c). Similar responses to relaxants were seen if the arteries were pre-contracted with noradrenaline rather than phenylephrine (n = 3-7; Additional file 1: Figure S5).

### Hepatic antioxidant expression

**Figure 6:**
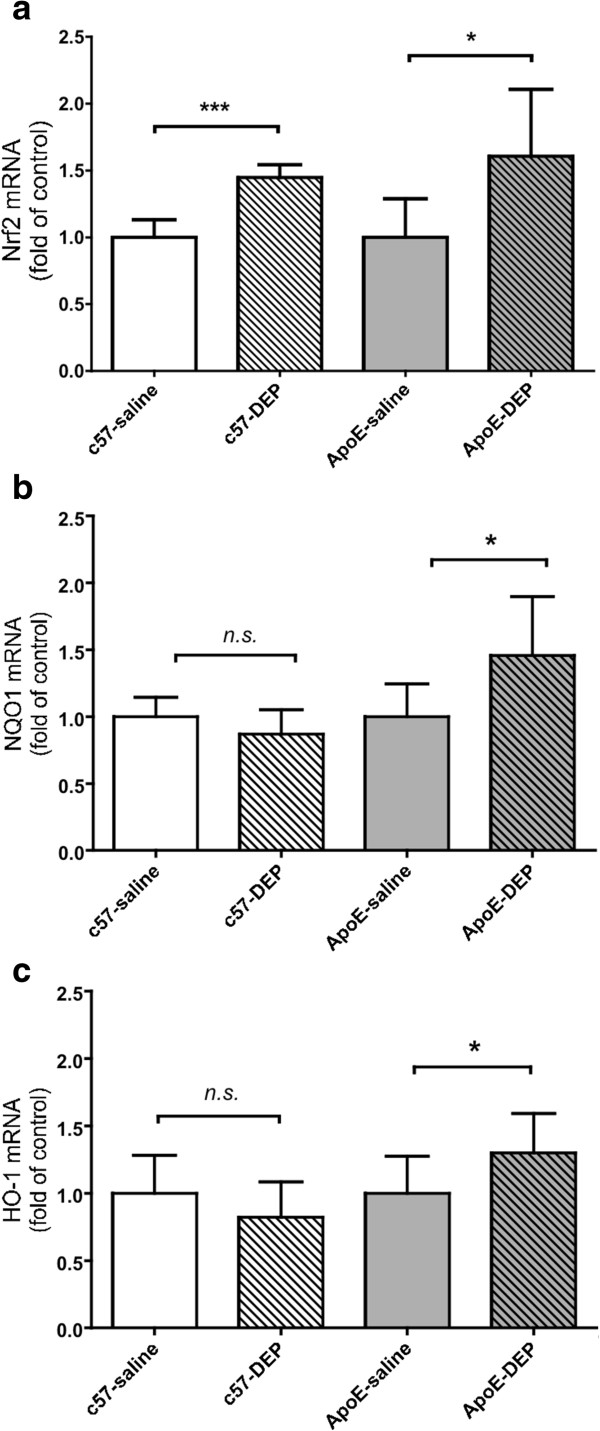
**Instillation of DEP increased expression of antioxidant genes in the liver, particularly in ApoE**^**−/− **^**mice. (a)** NF-E2-related factor-2 (Nrf2), **(b)** NAD(P)H-quinone oxidoreductase-1 (NQO1) and **(c)** hemeoxygenase-1 (HO-1). Effect of DEP shown as fold-increase in the expression in saline-treated mice. Mean ± S.E.M. (n = 5-8), ^n.s.^*P* > 0.05, *P < 0.05, ***P < 0.001, unpaired t-test comparing saline vs DEP. ApoE^−/−^ were fed Western diet for 8 weeks, whereas wild-type mice were fed normal chow.

Expression of antioxidants in liver was used to as an indicator of a response to a systemic oxidative stress. Instillation of DEP increased NF-E2-related factor-2 (Nrf2) expression in C57bl6 mice (*P* < 0.01; n = 5 for all; Figure 6). Exposure to DEP induced an increase in gene expression of Nrf2 (*P* = 0.023; n = 5 for all), NAD(P)H-quinone oxidoreductase 1 (NQO1; *P* = 0.034; n = 7 for C57bl6 groups, n = 5 for ApoE^−/−^ groups) and hemeoxygenase-1 (HO-1; *P* = 0.027; n = 8 for all) in ApoE^−/−^ mice (Figure 6).

## Discussion

We have here demonstrated that, in the murine apolipoprotein E deficiency model, instillation of DEP increased lesion size, produced more lesions per vessel and generated more buried fibrous caps. The proatherosclerotic effect was concomitant with pulmonary inflammation and systemic oxidative stress. We conclude that the particulate components of DE accelerate plaque development and are associated with a more complex plaque phenotype. The processes involved may contribute to the association between exposure to urban air pollution, atherosclerosis and acute myocardial infarction in man.

The influence of DEP was assessed using a recognised model of atherosclerosis at a period of extensive plaque expansion and remodelling [25,26]. Serial sections of artery were used to provide a surrogate marker of plaque volume and a sensitive approach to quantifying changes in plaque burden. We found that four weeks of repeated DEP exposure caused an almost doubling of plaque size from 32% to 59%. The 4-week exposure to DEP was consistent with pre-clinical investigations using concentrated ambient particles (CAPs) [8,9,27-30], whole DE [11,19] and, to a lesser extent, non-carbon nanoparticles [31]. These previous studies have been performed in several different animal models (ApoE^−/−^ mice, low density lipoprotein receptor Apo E double knockout mice and Watanabe heritable hyperlipidaemic rabbits) using either inhalation [8,10,11,19,30] or instillation [27,29,31]. The present study is the first to assess whether direct exposure to DEP increases atherosclerosis.

The marked increases in plaque size with DEP instillation are striking given the short duration of the exposure period. In the present study, long-term exposure has been emulated by administration of higher doses of particles over a shorter length of time. The instillation technique has been well validated [32-36] and the dose employed is similar to that of other studies using repeated instillations [37,38], and below those commonly used for single instillation exposures. While we cannot rule out that a proportion of the particulate administered deposits in the upper airways, studies have shown that the aspiration instillation technique to provide excellent delivery and dispersity of particulate throughout the alveoli [39,40]. The MPPD particle deposition model [41] estimates that the average 24-h instilled dose used here (assuming all DEP reaches the alveoli) is approximately 40-fold higher than the comparable alveolar deposition from a 24-h inhalation of 100 μg/m^3^ in man (adjusted for mice). It is important to highlight that these calculations are based on inhalation of particles with a primary particle diameter of that of diesel exhaust (~60 nm), whereas, urban levels of air pollution will contain a wide range of particulate sizes of which only a lower proportion of the larger particulates will penetrate past the tracheobronchial regions. Nevertheless, it is estimated that ~20% (6-41%; [42,43]) of the mass of PM_2.5_ is in the ultrafine range, and concentrations frequently reach 100 μg/m^3^ (a high percentage of which will originate from vehicle exhaust) for sustained periods in heavily polluted cities in both developed and developing nations. Furthermore, the airborne mass concentration of 100 μg/m^3^ represents a moderate/high PM levels over a 24-h period and does not take into account peaks in PM levels that regularly occur in cities, or the increased PM deposition produced by exercise such as brisk walking or cycling in urban environments. In comparison to a long-term continuous inhalation, the instillation regimen will unavoidably induce intermittent very high doses and dose-rate interspersed with long periods of non-exposure [44]. Nevertheless, the similarity of the current results with that of DE inhalation in ApoE^−/−^ mice [10,11,19,20,45] supports the contention that the biological pathways identified using the instillation protocol are both relevant and important. While a role for the gaseous component of DE cannot be excluded, our results suggest that the particulate component of DE alone is sufficient to enhance plaque formation. It is possible that the gaseous constituents could drive other responses leading to altered plaque composition [10] which we did not see in our study. The large magnitude of the pro-atherosclerotic effect of DEP will allow us to use this model in the future to explore which attributes and constituents of DEP (e.g. transition metals, polyaromatic hydrocarbons and quinones), and urban PM in general, are responsible for these effects.

Transient exposure to road-traffic has been linked to acute coronary events [46] and several pre-clinical investigations have suggested that exposure to atmospheric pollution increases markers of lesion vulnerability [8,10,11,27,29]. Urban PM [8,27,30,47,48], whole DE [11,19,20] or the gaseous components of DE [10], increase lipid content and inflammatory cells in plaques and the underlying vessel wall. Our results indicated that lipid and inflammatory cells increased in lesions from DEP-instilled mice, but only in proportion to lesion size.

While there was no change in the proportion of individual plaque constituents, or other typical markers associated with plaque vulnerability (e.g. MMPs), the complexity of the lesions in DEP-instilled mice was increased, with more lesions per section and more buried caps within lesions compared with controls. The results are similar to that previously observed in ApoE^−/−^ mice exposed to whole DE by inhalation: an effect that was prevented by addition of a cerium additive to the diesel fuel that decreased the number of particles in the exhaust [20]. It has been suggested that the presence of buried fibrous layers in the brachiocephalic artery are signs of plaque erosion or a previous plaque rupture [49,50]. The question as to whether buried fibrous layers are evidence of plaque rupture, or is merely a feature of the on-going development of a single plaque, remains a subject of debate [25,51]. Atherosclerotic lesions in ApoE^−/−^ mice do not rupture catastrophically, but similar changes in human lesions would be associated with increased plaque vulnerability [48,52]. Thus, these findings would suggest that the ability of DEP to increase plaque size and complexity are likely to be an important contributor to the cardiovascular manifestations of urban air pollution in man.

Previous investigations have suggested that increased atherosclerosis following exposure to DE or CAPs may be due to increased systemic inflammation [11,27-29], vascular dysfunction [8,19,30], or increased oxidative stress [9-11,30]. In the current investigation, DEP did not increase circulating concentrations of plasma lipids or plaque lipid content, therefore, the exacerbation of lesion formation cannot be attributed to altered lipid handling.

It has been hypothesised that inhaled particles exert their cardiovascular effects indirectly through the passage of inflammatory mediators from the lung to the systemic circulation [53]. DEP caused a clear lung inflammation characterised by infiltration of macrophages and neutrophils into the airways. Interestingly, there was an association with levels of lung inflammation and the size of atherosclerotic plaques. Other studies have failed to find an association between the ability of nanoparticles to induce pulmonary inflammation and their actions on a range of cardiovascular end-points (reviewed in [54]). Concentrations of acute phase proteins (CRP) in the blood were unchanged by DEP exposure. Although we cannot exclude transient surges in cytokines in response to the particulate, or the involvement of other cytokines (e.g. tumour necrosis factor alpha, interleukins, serum amyloid A3), we have not found consistent changes in any blood cytokine at either early (2 h) or late (24 h) time-points after DE exposure in man [14,15,55]. Future studies will address these possibilities in more detail. Additionally, in the present study DEP did not change the inflammatory cell content of atherosclerotic plaques, a finding similar to that after 40 days inhalation of CAPs [9]. Overall, while inflammatory pathways are likely to contribute to the cardiovascular effects of DEP, neither pulmonary nor systemic inflammation alone can account for the atherogenic actions of DEP.

Repeated instillation of DEP was not associated evidence of vascular dysfunction. Previous investigations have reported increased [8,19] or reduced [30] vascular contractility accompanying atherosclerosis following pollutant exposure. There have also been contradictory indications of normal [30] or impaired [8] acetylcholine-mediated relaxation. However, in the latter, the magnitude of the impairment was small and followed prolonged (>10 weeks) exposure and high-fat feeding. Alternatively, vasomotor impairment may be restricted to resistance arteries [56] and not the large conductance arteries where atherogenesis takes place. Our results suggest that vascular dysfunction is not required for DEP-induced acceleration of atherosclerosis.

The hepatic up-regulation of several protective antioxidant genes in response to DEP suggests a counter-regulatory response to the systemic pro-oxidative effects of DEP. We have previously shown that the Nfr2 pathway is also upregulated in systemic tissues in response to inhalation of ultrafine urban PM in ApoE^−/−^ mice [9] and whole diesel exhausts [45]. Interestingly, up-regulation of Nrf2 could trigger vascular proatherogenic effects as we have recently reported that systemic Nrf2 deletion inhibits rather than promotes atherosclerotic lesion formation in the aorta of ApoE null mice [57]. DEP itself can generate free radicals in solution [58], and oxidative stress is one of the most consistently proposed links between the pulmonary and systemic effects of particulate exposure [59]. Vehicle exhaust promotes lipid peroxidation in plasma lipoproteins and systemic tissues [60], consistent with recent studies where DE led to increased plasma levels of 8-isoprostanes, 12-HETEs, 13-HODEs, the development of dysfunctional prooxidative and proinflammatory high density lipoprotein (HDL) [45], and increased isoprostanes in urine [11]. Although we did not evaluate the status of lipid peroxidation in our study, we suggest that the systemic pro-oxidative effects of the particulates in DE could drive a significant portion of the proatherosclerotic actions of urban PM. Interestingly, antioxidant upregulation was especially notable in ApoE^−/−^ mice compared to wild-type mice. This observation adds to the growing evidence that animals/individuals with pre-existing vascular disease (or their risk factors), or diseases with associated cardiovascular complications (e.g. diabetes), may be particularly susceptible to the effects of air pollution [1,7,61,62].

## Conclusions

Exposure of the particulate constituents of diesel exhaust increases both the size and complexity of atherosclerotic plaques *in vivo*, and may therefore increase the susceptibility of plaques to rupture. The increased atherogenesis was not directly related to raised levels of systemic inflammation, changes in lipid handling or endothelial dysfunction, but was associated with pulmonary inflammation and systemic changes in antioxidant expression*.* Similar changes in man would be predicted to increase plaque vulnerability and rupture, helping to explain the associations between urban air pollution and acute myocardial infarction. These findings provide support for the implementation of strategies to remove vehicle-derived particulate emissions as a means to reduce the detrimental health effects of air pollution.

## Methods

Detailed Methods in Additional file 1.

### Animals, instillations and necropsy

All experiments were performed according to the Animals (Scientific Procedures) Act 1986 (UK Home Office). Adult male ApoE^−/−^ mice (N = 20) and the background strain (C57bl6 mice; N = 16) were purchased from Charles River (Margate, UK) aged 8–9 wks (20–25 g). C57bl6 mice were fed 8 weeks standard chow, whereas ApoE^−/−^ mice received a high fat ‘Western Diet’ (21% fat; Research Diets, New Brunswick, USA). This approach was used to allow a direct comparison of the effect of DEP on animals with no atherosclerosis with that of animals exhibiting large ‘complex’ atherosclerotic plaques, respectively.

For the final four weeks of feeding, mice underwent twice weekly instillation (oropharnyngeal aspiration) of 35 μg DEP (35 μL of 1 mg/mL; National Institute of Standards and Technology (NIST); SRM-2975; Gaithersburg, U.S.A.), to represent an average daily dose of 10 μg/mouse (see Discussion). This reference material is a commonly used source of DEP, allowing comparability with other researchers, and we have previously shown it has the capacity to generate superoxide free radicals in vitro, stimulate cultured macrophages and can directly impair arterial function [58,63]. The mean particle size of DEP in saline buffer prior to administration was 257 ± 46 nm (n = 6; dynamic light scattering, Brookhaven PS90 Particle Size Analyser; data not shown). Animals were sacrificed 3–4 days after their last instillation and blood (0.6-1 mL) withdrawn from the heart. Lungs were lavaged with sterile saline (3x 0.8 mL) to collect bronchoalveolar lavage fluid (BALF), and the carotid arteries, brachiocephalic artery, aortic arch, thoracic aorta and liver biopsies collected.

### Measures of pulmonary inflammation

BALF samples were centrifuged (180 *g*, 5 min, 4°C) and the cell pellets combined, resuspended, stained with Diff-Quik (Raymond A Lamb, London, UK) and prepared into cytocentrifuge smears for differential cell counts. The inflammatory cytokines interleukin-6 (IL-6), tumour necrosis factor alpha (TNF-α) and monocyte chemotactic protein-1 (MCP-1; CCL2/JE) were measured in the primary cell-free BALF by enzyme-linked immunosorbent assay (ELISA; R&D Duoset Systems; Patricell Ltd, Nottingham, UK).

### Assessment of atherosclerosis

The aortic arch and descending thoracic aorta were randomly selected from half of the mice from each treatment group. The artery was cut longitudinally and lipid-rich atherosclerotic plaques were stained *en-face* using Sudan IV.

Brachiocephalic arteries were fixed in formalin and histological sections were taken in triplicate at 100 μm intervals, beginning at the first section of artery with a fully intact media. Sections were stained with United States Trichrome (UST). The cross-sectional area of the plaque was measured and standardised to the medial area. The medial wall was chosen for standardisation rather than luminal area, as vessels could not be perfusion-fixed *in situ*, as they were also required for assessment of vascular function by myography (see below). A single mean value of atherosclerotic burden for each animal was calculated from the plaque size from each complete serial section throughout the brachiocephalic artery.

Plaque composition was assessed by both semi-quantitative scoring of fibrous matrix, foam cell and lipid content, and thickness of plaque cap, in UST-stained sections. Quantitative measurement of plaque constituents was performed using histology (collagen, fibrin) and immunohistochemistry (lipids, macrophages, smooth muscle cells, fibrinogen, metalloproteinases).

Plaque structure was assessed by counting distinct adjoining, or overlying, plaques within each section [20]. Additionally, the number of buried fibrous layers (defined as ‘a length of fibrous/cellular matter that completely bisects a lipid-rich regions of two overlying plaque sections [24]) was counted in each section throughout the artery and a mean value obtained. Samples were randomised before assessment and all scores independently verified by a second blinded assessor. See Additional file 1 for full details on methods used to assess atherosclerosis.

### Measurement of blood lipids, C-reactive protein and fibrinogen

Non-citrated blood was allowed to clot on ice (>2 hours, 4°C), centrifuged (10,000 rpm, 10 min) and used for colormetric measurement of cholesterol and triglycerides (Microgenics, St Albans, UK), and C-reactive protein (CRP) by ELISA (Innovative Research kits; Patricell, Nottingham, UK). Citrated blood was centrifuged (3,000 *g*, 15 min) to collect platelet-poor plasma for measurement of fibrinogen (Innovative Research ELISA kits; Patricell, UK).

### Vascular function

Segments (1–2 mm length) of the distal portion of the thoracic aorta were mounted in a multi-myograph system (610M; Danish Myo Technology, Aarhus, Denmark). Concentration-response curves were generated for phenylephrine (PE), the endothelium-dependent vasodilator, acetylcholine (ACh), and the endothelium-independent nitric oxide (NO) donor sodium nitroprusside (SNP). Only a single segment was used from each animal, and all dose response curves were performed in the same vessel. Drugs were washed out for >30 min, and prior dose response curves did not affect the responses to subsequent drugs.

### Expression of hepatic antioxidants

The expression of tissue mRNA for hemeoxygenase-1 (HO-1), NAD(P)H-quinone oxidoreductase 1 (NQO1), catalase, and NF-E2-related factor-2 (Nrf2) were measured by quantitative real-time polymerase chain reaction [9].

### Statistical analysis

Statistical comparisons were made using unpaired Student’s *t*-test or analysis of variance (ANOVA) with Bonferroni post-hoc tests where appropriate. Kruskal-Wallis or Mann–Whitney tests were used for non-parametric or ordinal level data (1–5 scoring of plaque components). Two-sided *P* < 0.05 was taken as statistically significant.

## Abbreviations

ACh: Acetylcholine; ANOVA: Analysis of variance; ApoE−/−: Apolipoprotein E knockout; BALF: Bronchoalveolar lavage fluid; CAPs: Concentrated ambient particles; CRP: C-reactive protein; DE: Diesel exhaust; DEP: Diesel exhaust particulate; ELISA: Enzyme-linked immunosorbent assay; HO-1: Hemeoxygenase-1; IL-6: Interleukin-6; MCP-1: Monocyte chemotactic protein-1; MMP: Matrix metalloproteinase; NIST: National Institute of Standards and Technology; NQO1: NAD(P)H-quinone oxidoreductase 1; Nrf2: NF-E2-related factor-2; NO: Nitric oxide; PE: Phenylephrine; PM: Particulate matter; S.E.M.: Standard error of the mean; SMA: Smooth muscle actin; SNP: Sodium nitroprusside; TNF-α: Tumour necrosis factor alpha; UST: United States Trichrome.

## Competing interests

The authors declare that they have no competing interests.

## Authors’ contributions

MRM designed the study, carried out the experimental work, carried out the statistical analysis and drafted the manuscript. SGM carried out the experimental work, and assisted with the data and image analysis. RD assisted with the particle instillations and pulmonary assays, and contributed to the drafting of the manuscript. AOL carried out the antioxidant expression work and analysis. JAA contributed to the interpretation of the data and helped draft the manuscript. CAS contributed to the drafting of the manuscript. NLM contributed to the drafting of the manuscript. KD participated in the design and interpretation of the study, and helped to draft the manuscript. DEN participated in the design and interpretation of the study, and helped to draft the manuscript. PWFH participated in the design and interpretation of the study, and helped to draft the manuscript. All authors have read and approved the final version of the manuscript.

## Supplementary Material

Additional file 1 On-line supplementary information. Click here for file
