# Supplementary material for: Diesel exhaust particulate increases the size and complexity of lesions in atherosclerotic mice
Source: Part Fibre Toxicol. 2013 Dec 11;10:61. doi: 10.1186/1743-8977-10-61 (PMC3907045; doi:10.1186/1743-8977-10-61)
Supplement: Additional file 1 — On-line supplementary information. [file 1743-8977-10-61-S1.doc]

**Miller et al.** Diesel exhaust particulate promotes atherosclerosis

**On-line Supplementary Information**

*Changes to manuscript marked in red. Re-ordering of references shown as* ***[****No****]*** *with reference details in* ***black*** ***text*** *in bibliography. New references shown as* ***[****No****]****, inserted will be detailed in* ***red text*** *in bibliography.*

**Methods in full**

*Drugs and reagents*

All drugs were obtained from Sigma Ltd. (Poole, U.K.) or VWR International (Lutterworth, U.K.) and dissolved/diluted in distilled H_2_O, saline or Krebs buffer (composition in mM: 118.4 NaCl, 25 NaHCO_3_, 11 glucose, 4.7 KCl, 1.2 MgSO_4_, 1.2 KH_2_PO_4_, 2.5 CaCl_2_) unless stated otherwise.

*Animals*

All experiments were performed according to the Animals (Scientific Procedures) Act 1986 (U.K. Home Office). Adult male ApoE knockout mice (ApoE^-/-^; Apoe^tm1Unc^/J; 20 mice in total) and the background strain C57bl6 mice (C57BL/6J; 16 mice in total) were purchased from Charles River (Margate, UK) at a starting age of 8-9 wks (20-25g). Animals were given 1 week to acclimatise with food (RM1, Special Diet Services, Essex, UK) and water given *ad libitum*. At the end of this week C57bl6 mice were continued to be fed on standard chow, whereas ApoE^-/-^ mice were placed on high fat ‘Western’ diet’ (21% fat; Research Diets, New Brunswick, USA for 8 weeks until the time of sacrifice, to accelerate the development of atherosclerotic lesions. This approach was used to allow a direct comparison of the effect of DEP on animals with no atherosclerosis with that of animals exhibiting large ‘complex’ atherosclerotic plaques.

*Administration of diesel exhaust particulate (DEP)*

DEP (National Institute of Standards and Technology; NIST; SRM-2975; Gaithersburg, U.S.A.) suspensions were prepared daily (1 mg/mL stock solution) in sterile saline (0.9% NaCl), followed by 5 min probe sonication (100% power, FB11002, Fisher Scientific, Loughborough, UK) on ice to minimise particle aggregation. This reference material is a commonly used source of DEP, allowing comparability with other researchers, and we have previously shown it has the capacity to generate superoxide free radicals in vitro, stimulate cultured macrophages and can directly impair arterial function .

For the final four weeks of feeding, mice were instilled into the lung twice per week with 35 μg DEP by oropharyngeal aspiration , to represent an average dose of 10 μg/day. Mice were anaesthetised using isofluorane inhalation (~3 minutes; Merial, Essex, UK), the tongue pulled forward with forceps and a bolus suspension (35 μL of 1 mg/mL DEP or saline vehicle) pipetted onto the oropharynx. The nares of the rodent were occluded using the forefinger and thumb, and the tongue held forward until total aspiration occurred. Animals were then placed back in their cage and monitored until return to normal behaviour (<5 min). Pulmonary instillation of DEP was chosen as the exposure method; to administer only the particulate components of diesel exhaust, to ensure the complete delivery of the dose to the lungs, and establish this model as a basis for future experiments looking at the effects of fractionated DEP. The feeding and instillation protocol was designed to provide repeated exposure to DEP across a period with a high degree of atherosclerotic remodelling , using a number and frequency of instillations deemed to be appropriate for the welfare of the animal. The mean particle size of DEP in saline buffer prior to administration was 257±46 nm (n=6; dynamic light scattering, Brookhaven PS90 Particle Size Analyser; data not shown).

*Necropsy and bronchoalveolar lavage*

Animals were sacrificed 3-4 days after their last instillation and were not fasted prior to sacrifice. Mice were anaesthetised by intraperitoneal injection of Avertin (200 μL of 1.25% tribromoethanol /10 g body weight), the thoracic cavity was exposed and blood (0.6-1 mL) withdrawn directly from the heart using a 23 gauge needle. Half of the blood collected was dispensed into tubes containing sodium citrate (to make a final concentration of 0.32 %), and the other half was collected into tubes with no anticoagulant. Tubes were inverted slowly and placed on ice.

The lungs were cannulated via a small incision in the trachea and lavaged with a 0.8 mL volume of sterile saline. This primary lavage was retained in a separate tube for analysis of a bronchoalveolar lavage fluid (BALF) profile for cytokines. Subsequently, the lungs were lavaged with 2 further 0.8 mL volumes of sterile saline. In a small subset of animals the lungs were not lavaged and instead fixed for histological analysis. Lungs were perfusion fixed with ~2 mL of methacarn fixative (60% methanol, 30% chloroform and 10% glacial acetic acid) and the lungs allowed to slowly inflate under gravity.

The carotid arteries, brachiocephalic artery, aortic arch and thoracic aorta were removed in a single piece and placed in ice-cold Krebs Buffer until isolation of individual arteries. Tissues for histological analysis were fixed in 10% neutral-buffered formalin.

*Measures of pulmonary inflammation*

BALF samples were centrifuged (180g, 5 min, 4°C) and the supernatant removed. The cell pellets from each lavage were combined then resuspended in phosphate-buffered saline (PBS; 1 mL). Total cell numbers were counted and cytocentrifuge smears were prepared and stained with Diff-Quik (Raymond A Lamb, London, UK) for differential cell counts to be assessed. Three hundred cells per slide were counted. The inflammatory cytokines, interleukin-6 (IL-6), tumour necrosis factor alpha (TNF-α) and monocyte chemotactic protein-1 (MCP-1; CCL2/JE) were measured in the primary cell-free BALF preparation by enzyme-linked immunosorbent assay (ELISA; R&D Duoset Systems; Patricell Ltd, Nottingham, UK) according to the manufacturer’s instructions. A Dynatech MRX microplatereader (Thermo Life Sciences, Basingstoke, UK) was used to measure absorbance of ELISA plates at the specified wavelength and analyse standards.

En-face *staining of plaques with Sudan IV*

Following removal of a ring of distal aorta for myography (see below), the aorta (arch and descending thoracic) randomly selected from half of the mice from each treatment group was cleaned thoroughly of adventitial fat (vascular tissue from the remaining mice was used for the development of additional assays, which, due to technical difficulties, are not reported). The artery was cut longitudinally, and pinned out on Sylgard® silicone elastometer (World Precision Instruments, Stevenage, UK) with the luminal surface uppermost. Lipid-rich atherosclerotic plaques were stained using Sudan IV (0.5% in 1:1 acetone: 70% ethanol; 15 min) followed by immersion in 80% ethanol (20 min) and differentiating in running tap water (1 h).

*Histological preparation of arteries*

The brachiocephalic artery was isolated, cleaned of connective tissue and fixed in formalin (20 h). Arteries were then embedded in low-melting point agar to aid orientation, before being further embedded in paraffin. Five micrometer sections were taken in triplicate at 100 μm intervals, beginning at the first section of artery with a fully intact media . Sections of carotid artery and thoracic aorta were taken from some animals and processed in the same way at 500 μm intervals.

A single replicate of all sections was stained with United States Trichrome (UST) , and key sections were chosen for staining for Martius Scarlet and Blue (MSB) , Masson’s Trichrome , picrosirius red or immunohistochemsitry as follows. For macrophage (MAC2) staining, a rat anti-mouse primary antibody was used (1/12000; CL8942AP, VH Bio, Gateshead, UK) with rat IgG (1/12000; I-400, Vector Labs, Peterborough, UK) as a negative control, followed by a goat anti-rat IgG biotinylated secondary antibody (BA-9400, Vector Labs). For smooth muscle actin (SMA) staining, a mouse anti-mouse primary antibody (1/400; A2547, Sigma Aldrich), with mouse ascites (1/400; M8273, Sigma) as a negative control, followed with a goat anti-mouse biotinylated secondary antibody (1/400; BA-9200, Vector Labs). Both SMA and MAC2 sections were incubated with extravidin-peroxidase solution (1/200). For MMP-2 and MMP-9 staining, endogenous peroxidase was quenched by incubation in 1% H_2_O_2_ in methanol. Heat-induced epitope retrieval was then performed using Tris-EDTA, heated 30 min in a microwave (1200W), and blocked with 20% normal goat serum. Mouse anti-MMP-2 (25ug/ml; ab7032, Abcam, Cambridge, UK) or mouse ascites as a negative control (dilution 1/200, M8273, Sigma, Dorset, UK) were incubated overnight before addition of goat anti-mouse IgG secondary antibody (dilution 1/400; BA9200, Vector Labs, Peterborough, UK). Mouse anti-MMP-9 (1/500 dilution; ab3889, Abcam, Cambridge, UK) or rabbit IgG as a negative control (1/200 dilution; X0903, Dako, Cambridge, UK) were incubated overnight at 4^o^C, before addition of goat anti-rabbit biotinylated secondary antibody (PK-4001; Vector Labs, Peterborough, UK). For fibrinogen staining, sections were treated with proteinase K (20 μg/mL), then blocked with 20% goat serum (in 1% bovine serum albumin in phosphate saline buffer). A rabbit anti-human primary antibody (1/800 dilution; A0080, Dako, Ely, UK) with cross-reactivity for the mouse and rabbit IgG (1/800; X0903, Dako) as a negative control, were followed with a goat anti-rabbit biotinylated secondary antibody (PK-4001, Vector Labs, Peterborough, UK).

In all cases, slides were incubated with avidin biotin complex (ABC; PK-4001, Vector Laboratories, Peterborough, UK), then detected by incubation with 0.05% 3,3’-diaminobenzidine (DAB; SK-4100; Vector Labs, Peterborough, UK), until staining was detected. Nuclei were counterstained with Harris’ hematoxylin.

*Assessment of plaque size, number and vulnerability*

Surface coverage of Sudan IV-stained plaques is expressed as a percentage of the total intimal surface area of the thoracic aorta, from the aortic root to the intercept with the diaphragm.

Brachiocephalic arteries were used for assessment of plaque volume, by analysis of serial cross-sections (every 100 μm) throughout the entire length of this artery. Plaque size was measured, then standardised to the area of the medial wall for each section, before a mean plaque size was obtained for the entire artery, for every animal. The medial wall was chosen for standardisation rather than luminal area, as vessels could not be perfusion-fixed *in situ*, as they were also required for assessment of vascular function by myography (see below).

Plaque composition was assessed by both semi-quantitative scoring of UST-stained sections and fully quantitative measurement of individual constituents within a single section chosen from the central region of the brachiocephalic artery. For scoring, each section was given a score between 0-5 for each of the following categories:

**Fibrous matter (elastin/collagen) Plaque cap**

0 = no fibrous areas 0 = no cap

1 = <20% plaque is fibrous 1 = cap layer appears to be a single layer of cells

2 = 20-50% plaque is fibrous 2 = cap 2-3 cells thick

3 = 50-80% plaque is fibrous 3 = cap 4-5 layers thick

4 = >80% plaque is fibrous 4 = cap >6 layers thick in most places of plaque

5 = 100% fibrous 5 = cap indistinguishable from rest of plaque

**Foam cell content Lipid cavities**

0 = no distinct foam areas 0 = no holes in plaque

1 = <20% foam cells 1 = few small holes in plaque

2 = 20-50% plaque is foam cells 2 = many small holes/few medium holes in plaque

3 = 50-80% plaque is foam cells 3 = large central core/many medium holes in plaque

4 = >80% plaque is foam cells 4 = very large central core/many large holes in plaque

5 = 100% foam cells 5 = <80% plaque is hole

A mean value of all sections calculated for each category and an overall score for ‘plaque vulnerability’ using the formula:

Vulnerability Score = (foam cell content + lipid content)

(fibrous content + cap thickness)

Low values are taken to represent a region of ‘stable’ atherosclerosis, and a value of 10 represents an area of atherosclerosis containing plaques believed to be susceptible to rupture (, based on the definitions in ).

A fully-quantitative measure of specific plaque components was also carried out following (immuno)histological staining of a single section of the brachiocephalic artery from one of the three sections exhibiting the greatest % plaque area. Sections were imported into Adobe Photoshop v11.0, and a colour range was selected from three randomly chosen positively stained sections, which was then used to identify positively stained plaque components from all subsequent slides. Immunohistological staining was used to identify components representing the presence of inflammatory cells (MAC-2) and smooth muscle cells (SMA). Picrosirius red was used to identify areas of collagen, and UST-stained sections were used to identify plaque lipids, on the assumption that intra-plaque areas showing no staining were areas previously containing lipids before the fixing procedure (preliminary experiments with frozen sections without formalin/ethanol fixation, showed that these cavities stained positively using the lipid stain oil-red-O; see also ). Positively identified areas were expressed as a percentage of the total plaque area, the value of which was used to calculate an additional score of plaque vulnerability, using the formula:

Vulnerability Index = (lipid cavities + MAC2 staining)

(SMA staining + collagen staining)

A score of 0 is considered to represent a ‘stable’ plaque, and a maximum value of 200 represents an extremely ‘unstable’ plaque .

The presence of buried fibrous caps is taken as general marker of plaque complexity either from the ongoing to read development of a single plaque or the merging of two separate sites of plaque growth . However, it has also been suggested that buried fibrous caps represent the growth of a new plaque over a site of a previous plaque rupture . These possibilities were considered by counting the number of potentially distinct plaques within an artery (either existing separately or adjoining with a clear fibrous divide indicating the merging of two separate plaques) and the number of buried fibrous caps within each section, whereby a buried fibrous cap was defined as “a length of fibrous/cellular matter that completely bisects lipid-rich regions of two overlying plaque sections”.

All samples were randomised before assessment and scores independently verified by a second blinded assessor.

*Blood lipids, inflammatory markers and fibrinolytic pathways*

Non-citrated blood was used for the measurement of cholesterol, triglycerides and C-reactive protein (CRP). Blood was allowed to clot on ice for >2 hours, before being centrifuged (10,000 rpm, 10 min) for the collection of serum, which was frozen (-80^o^C) until the time of assay. Cholesterol and triglycerides were measured by absorbance at 500 and 600 nm following reaction of serum (2 μL) with the appropriate detection reagents (200 μL; TR13923, TR22923; Microgenics, St Albans, UK) for 5 min at 37^o^C, in parallel with their standards (cholesterol: 0.97-38.8 mM; triglycerides: 0.28-7.91 mM; Microgenics). Serum CRP was measured by ELISA (Innovative Research IR200001; Patricell, Nottingham, UK) according to manufacturer’s instructions, following a 1 in 20 dilution of samples in the diluent provided. Citrated blood was centrifuged (3,000g, 15 min) to collect platelet-poor plasma. Plasma was diluted where appropriate and analysed for fibrinogen (1 in 1000 dilution), total t-PA antigen and t-PA activity (neat plasma, or 1 in 2 dilution if required) by ELISA (Innovative Research MFBGNKT, MTPAKT-TOT and MTPAKT, respectively; Patricell, UK) according to the manufacturer’s instructions.

*Vascular function using myography*

Segments (1-2 mm length) of the distal portion of the thoracic aorta were cleaned of connective tissue and mounted on 40 μm wires in a multi-myograph system (610M; Danish Myo Technology, Aarhus, Denmark) in Krebs buffer bubbled with 5% CO_2_/95% O_2_ at 37^o^C. A baseline tension of 8 mN was gradually applied over 10 min and vessels were allowed to equilibrate for a further 30 min. Preliminary experiments showed that this tension produced optimal contraction and dilatation responses. Data from force transducers were processed by a MacLab/4e analogue-digital converter displayed through Chart^TM^ software (AD Instruments, Sussex, UK).

Vessel viability was confirmed by a contractile response to serial addition of high K^+^Krebs (Krebs with substitution of 4.7 mM NaCl and 118.4 mM KCl) with 10 μM noradrenaline (NA), K^+^Krebs+NA, K^+^Krebs alone, NA alone and finally K^+^Krebs+NA. Concentration-response curves to phenylephrine (PE; 1 nM – 10 μM) were obtained and a concentration that produced 80% maximum contraction (EC_80_; ~1 μM) was chosen for each individual rat aortic ring. Noradrenaline (NA) was also used for preconstriction (EC_80_ = 0.5 μM) to confirm that the type of preconstrictor agent did not affect vasodilator responses between groups. Following precontraction, cumulative concentration-response curves were obtained for the endothelium-dependent vasodilator, acetylcholine (ACh; 1 nM to 10 μM) and the endothelium-independent NO donor sodium nitroprusside (SNP; 0.1 nM to 1 μM). Only a single segment was used from each animal, and all dose response curves were performed in the same vessel. At least 30 min washout was allowed before application of subsequent drugs and prior dose response curves did not affect the responses to subsequent drugs (data not shown). In any experiment where there were indications of loss of vessel performance (e.g. differences between contraction to EC_80_ doses of contractile agents in subsequent concentration-response curves, or failure of tone on precontraction) the data were excluded.

*Quantitative PCR for expression of hepatic antioxidants*

Due to the limited availability of atherosclerotic vessels and technical difficulties with assays we were unable to test for oxidative stress in vascular tissue. Instead, expression of antioxidants in liver was used to as an indicator of a response to a systemic oxidative stress. Antioxidant expression was assessed in the liver as described . Total RNA was extracted from liver using TRIzol Reagent (Invitrogen, San Diego, CA, USA). RNA (~2 µg) was transcribed into cDNA using a high-capacity cDNA reverse transcription kit (Applied Biosystems, CA, USA). Expressions of tissue mRNA of hemeoxygenase-1 (HO-1), NAD(P)H-quinone oxidoreductase 1 (NQO1), NF-E2-related factor-2 (Nrf2) and β-actin were measured by quantitative real-time polymerase chain reaction (qPCR) using specific primers (Table S1). The qPCR reactions were performed in triplicate for each sample on a LightCycler® 480 system (Roche, CA, USA) using LightCycler® 480 SYBRGreen I Mastermix (Roche). The reactions consist of 900 nM (final concentration) of each primer and 1 µg of cDNA template in a total reaction volume of 12 µl. The reactions were performed as follows: 95^o^C for 3 min, 40 cycles of 95^o^C for 15 sec, 58-64^o^C for 30 sec and 72^o^C for 30 sec. A standard curve was prepared by making serial dilutions of a pooled sample of cDNA and gene expression was normalized to β-actin and expressed as fold of control.

Though the RT-qPCR was quantified by SYBR Green, the data have been validated using specific probes from the ROCHE Universal Probe Library (data not shown). The specificity of the PCR primers used was examined on agarose gels to confirm that the only single band of the expected size corresponding to the specific amplified product was obtained. Melting points and amplification curves from all the samples were used to confirm that the specific product was amplified and that there were no primer-dimer effects or contaminations. Standard curves were constructed using pools of cDNA from the different samples and all the values fell within these standards.

*Statistical and data analysis*

All data are expressed as mean±SEM. Spectrophotometer readings from ELISAs were converted using an Akima curve plotted from the standards at concentrations suggested in the ELISA manufacturer’s instructions. All standards and unknown samples were made in duplicate (with the exception of t-PA measures that were run in singlicate due to the limited volume of plasma available). Vasodilator responses are expressed as percentage of the precontraction, where positive values represent vasodilatation and 100% vasodilatation represents a complete abolition of PE-induced tone.

Statistical comparisons were made using unpaired Student’s *t*-test or one-way analysis of variance (ANOVA), with Bonferonni post-hoc tests of selected comparisons where appropriate. Comparisons of concentration-response curves were made using two-way ANOVA. Kruskal-Wallis or Mann-Whitney tests were used to make comparisons non-parametric data (where there was evidence of a non-Gaussian distribution) and ordinal levels of data (1-5 scoring of plaque components). *P*<0.05 was accepted as statistically significant.

**Additional Results**

*DEP instillations were not associated with changes in body weight*

ApoE^-/-^ mice fed Western diet had marginally higher body weights than C57bl6 mice fed standard chow (28.9±0.7g and 27.8±0.4g respectively), although this difference did not reach statistical significance (*P*=0.0525, Mann-Whitney test, n=16-20). These levels were not significantly influenced by instillation of DEP (*P*=0.11; Kruskal-Wallis followed by Dunn’s post-hoc tests, n=8-10 for all; Fig S1).

*Alternative methods to assess the size of atherosclerotic plaques*

DEP increased the size of atherosclerotic plaques in the brachiocephalic artery, when plaque size was expressed as a percentage of the area of the media and a mean value taken for the entire length of artery (Fig 2, main document). This difference was also apparent if lesion size was standardised as a percentage of the area of vessel lumen (*P*=0.007, unpaired t-test, n=6-8; Table S2). Large lesions developed throughout the entire length of the brachiocephalic artery of ApoE^-/-^ mice. If lesion size was taken only from the section with the largest lesion only (a common, but less sensitive, approach to detect differences in atherosclerotic burden), DEP was found to increase this measure of plaque size to a similar extent (*P*=0.042, unpaired t-test, n=7-9; Table S2). No evidence of plaques was found in sections taken from the middle of the carotid or the distal portion of the descending thoracic aorta.

*Phenotype of atherosclerotic plaques*

Lesions varied markedly in size and composition, from thin regions of foam cell formation along the intimal surface, to large lesions protruding into the lumen of the vessel, composed of foam cells and lipid cavities surrounded by a thin fibrous cap, to more complex lesions exhibiting layers of smooth muscle and foam cells, crystal-shaped cholesterol cavities and thick localised matrices of fibrous tissue. Plaques were categorized into those that were ‘largely foam cells’ (>70% foam cells and lipids), largely fibrous matter (>70% elastin and collagen-rich tissue) or those of a ‘mixed appearance’ (30-70% elastin or foam cells). There was no obvious trend in the effect of DEP on the frequency of each plaque type, and accordingly no significant differences between saline and DEP for each category (P>0.05; one-way ANOVA followed by Bonferroni post-hoc tests; n=5-6; Fig S2a).

The susceptibility of a plaque to rupture was estimated by assessing individual plaque components that are likely to influence plaque stability. Initially, a scoring system was assigned to plaque components likely to improve stability (fibrous content and thickness of plaque cap) and those associated with likelihood of rupture (lipid content and foam cell content). None of these factors were different between the DEP-instilled group in comparison to the saline-treated animals (*P*>0.11, Mann-Whitney test; n=5 for all), either alone or when combined into a composite score of plaque vulnerability (*P*=0.27, Mann-Whitney test, Fig S2b).

Key sections from brachiocephalic arteries were chosen for identification of specific constituents. There was generally a greater amount of all components of plaques from DEP-treated mice (Fig S2c), in accordance to the greater plaque size of this group, with MAC-2 (*P*=0.027) and collagen (*P*=0.045) staining being significantly different from the saline-treated group. However, when expressed as a percentage of the total plaque area, DEP instillation had no significant effect on the percentage of plaque with MAC-2-stained foam cells (P=0.40), lipid cavities (P=0.26), smooth muscle cells (P=0.35) or collagen (P=0.22; all unpaired t-tests n=7-9; Fig S2d). Plaque vulnerability, calculated from a composite of these scores, was also not significantly changed by DEP instillation (P=0.064, unpaired t-test, n=7-9).

*Plaque matrix metalloproteinase expression*

Sections were stained for matrix metalloproteinase (MMP) types 2 and 9 as these isoforms are believed to play a role in the breakdown of extracellular matrix of the atherosclerotic plaque leading to plaque vulnerability . MMP2 was detected in the media wall of the blood vessel, but was particularly prevalent in plaques, especially in foam cell-rich lesions (Fig S3a). DEP treatment had no effect on MMP-2 staining in comparison to saline treated animals (*P*=0.48, unpaired *t*-test, n=5-9; Fig S3b). MMP-9 staining was weak and diffuse (Fig S3c), and was not significantly different between plaques from DEP-treated animals compared to saline-treated (*P*=0.48, unpaired *t*-test, n=4-9; Fig S3d).

*Examination of plaques for blood constituents within lesion*

The presence of fibrin within plaques may indicate the breakdown of blood components either from the formation of a plaque over a thrombus at a site of previous rupture or the formation of new blood vessels within a plaque . Both MSB and Masson’s trichrome were used to detect fibrin, however, positive (red) staining was only observed in a few slides, the areas of which were very small and not associated with buried fibrous layers or eroded cap surfaces. There was no indication that the frequency of staining was greater in plaques from DEP-instilled animals, compared to that of saline-instilled animals (Fig S4a,b). Sections were also stained for fibrinogen, which was readily detected in plaques, particularly in areas associated with foam cells (Fig S4c). There was no significant difference in the percentage of the plaque staining for fibrinogen between saline or DEP-treated mice (*P*=0.08, unpaired t-test, n=5-6; Fig S4d).

*Quantification of inflammatory markers in BALF and blood*

There was a tendency for DEP instillation to increase levels of the cytokines IL-6, TNFα and MCP-1 (CCL2/J6) in BALF from ApoE^-/-^ mice (*P*>0.13, unpaired *t*-tests; n=4-7), although the protein levels were very low; around or below the levels of the lowest standard (Table S3).

Blood fibrinogen levels can be indicative of imbalances in thrombus formation and thrombolysis, but it is also an acute phase protein the levels of which are closely associated with other markers of systemic inflammation . Levels of fibrinogen in blood were unaffected by DEP instillation (both *P*>0.20; one-way ANOVA followed by Bonferroni post-hoc tests; n=7-8 for all; Table S3). Analysis of serum CRP levels showed a trend towards a slightly reduced CRP levels in C57bl6 mice following DEP administration (*P*=0.02; one-way ANOVA comparing all groups; Table S3), however, Bonferroni post-hoc tests did not find statistically significant differences between any groups. Plasma levels of t-PA, both total antigen and active t-PA, were below detection limits.

*Vasomotor function: preconstrictor agents*

Vasodilator responses in isolated vessels can be influenced by the type of agent used to precontract vessels and the level of precontraction induced . For this reason, responses to acetylcholine and sodium nitroprusside were carried out in vessels precontracted with EC_80_ NA as well as EC_80_ PE. The pattern of vasodilator responses was similar for both types of preconstrictor used (Fig S5). There was no difference in the responses in noradrenaline-contracted vessels from saline-treated and DEP-treated animals in either C57bl6 or ApoE^-/-^ mice (*P*>0.05, two-way ANOVA, n=3-7; Fig S5).

**Supplementary Figures & Tables**

**Table S1**. qPCR primer sequences

| Genes | Sequence |
| --- | --- |
| β-actin | Forward: AGCCATGTACGTAGCCATC  Reverse: CTCTCAGCTGTGGTGGTGA |
| HO-1 | Forward: CACGCATATACCCGCTACCT  Reverse: CCAGAGTGTTCATTCGAGA |
| NQO1 | Forward: TTCTCTGGCCGATTCAGAGT  Reverse: GGCTGCTTGGAGCAAAATG |
| Catalase | Forward: GAGACCTGGGCAATGTGAT  Reverse: GTTTACTGCGCAATCCCAAT |
| Nrf2 | Forward: CTCGCTGGAAAAAGAAGTG  Reverse: CCGTCCAGGAGTTCAGAGG |

**Figure S1**


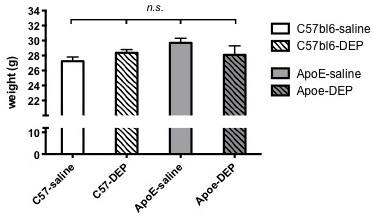


**Figure S1.** Weight of mice at time of sacrifice. There were no statistically significant differences between C57bl6 or ApoE mice (*P*>0.05, Mann-Whitney test, n=16, 20 respectively), or between saline vs DEP-instilled animals (*P*>0.1, Kruskal-Wallis followed by Dunn’s post-hoc tests; n=8-10 for all groups). ApoE^-/-^ were fed Western diet for 8 weeks, whereas wild-type mice were fed normal chow.

**Table S2.** Cross-sectional area of atherosclerotic plaques in brachiocephalic arteries, obtained by three different methods of analysis.

Group: C57bl6* ApoE^-/-^

Treatment: saline DEP n saline DEP *P* n

entire artery**

% of media 0.8±0.5 0.4±0.2 3-5 31.7±7.1 59.3±9.7 0.017 7-9

entire artery**

% of lumen - - - 18.5±3.9 64.0±17.7 0.007 6-8

largest section

% of media - - - 49.6±10.7 86.8±18.2 0.042 7-9

*Single method of plaque assessment in C57bl6 mice only due to absence of clear plaques in these animals

** Values obtained from analysis of serial sections at 100 μm intervals for each animal. :=4.4 sections per animal.

P-values shown are comparison of saline versus DEP, unpaired t-tests.

**Figure S2**


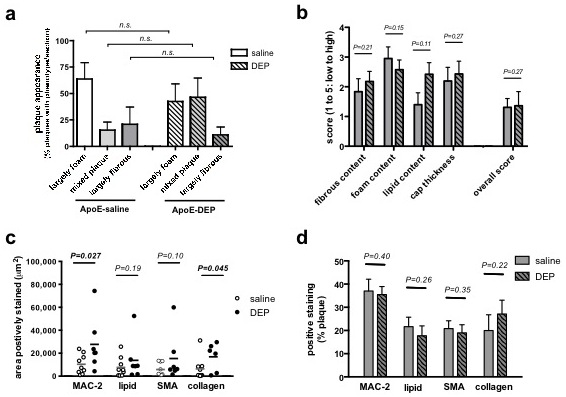


**Figure S2.** Plaque phenotype and composition **(a)** Exposure to DEP did not alter the proportion of different types of lesion developing in brachiocephalic arteries ^n.s.^*P*>0.05, Bonferroni post-hoc test, following one-way ANOVA. **(b)** Plaque characteristics used for calculation of vulnerability score. 0=low, 5=high. *P*-values calculated from Mann Whitney test of saline versus DEP. **(c)** Area (μm^2^) staining positive for different plaque components. Saline (open circles, mean=grey bar), DEP (filled circles, mean=black bar). **(d)** Percentage of the lesion staining positive for different plaque constituents. *P*-values calculated from unpaired t-tests of saline versus DEP. Date are Mean+S.E.M. For (a,b) Mean value obtained from analysis of serial sections at 100 μm intervals for each animal; Mean number of sections per group = 5.0±0.8 for ApoE-saline (n=6), 4.0±1.0 for ApoE-DEP (n=5). For (c,d) a single section from each animal used for constituent staining; n=9 for ApoE-saline, n=7 for ApoE-DEP.

**Figure S3**


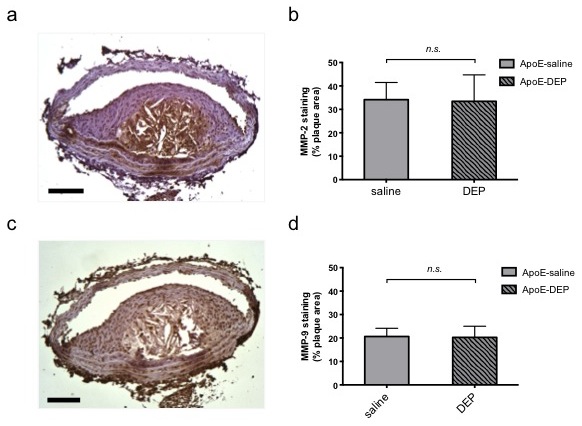


**Figure S3.** Instillation of DEP did not increase levels of metalloproteinases (MMP) in plaques. **(a)** MMP-2 and **(c)** MMP-9 immunohistochemical staining (brown regions). Scale bar = 100 μm. The proportion of **(b)** MMP-2 and **(d)** MMP-9 in plaques were not significantly different between saline and DEP-treated animals. A single section from each animal used for constituent staining. Mean±S.E.M n=9 for ApoE-saline, n=4 for ApoE-DEP. ^n.s.^*P*>0.05 by Student’s unpaired t-test.

**Figure S4**


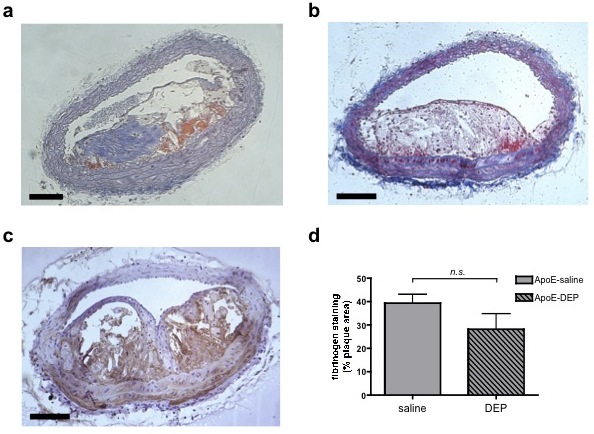


**Figure S4.** Identification of fibrin or fibrinogen in atherosclerotic lesions using different staining tehniques. **(a)** Martius Scarlet and Blue (MSB; red staining may represent fibrin). **(b)** Masson’s Trichrome (red staining may represent fibrin). There was no evidence of fibrin associated with buried fibrous layers or within the shoulder regions of plaques. **(c)** Fibrinogen (brown stain), Scale bar = 100 μm. **(d)** DEP did not significantly affect the proportion of positive staining with the fibrinogen antibody within plaques. A single section from each animal used for constituent staining. ^n.s.^*P*>0.05, unpaired t-tests. Mean±S.E.M. (n=5-6).

**Table S3 –** Measurement of inflammatory, coagulability and fibrinolytic markers in bronchoalveolar lavage fluid or blood plasma/serum at time of sacrifice.

Group: C57bl6 ApoE^-/-^

Treatment saline DEP *P* n saline DEP *P* n__

**BALF** (all pg/ml)

IL-6 64±17 72±5 0.33 4 22±16 50±15 0.13 4-6

TNFα 57±7 53±16 0.41 4-5 24±10 33±10 0.25 6-7

MCP-1 (CCL2/JE) 4.8±1.0 3.5±1.5 0.23 4-5 2.5±0.5 3.5±0.9 0.37 4-5

**Blood** (all ng/ml, except fibrinogen: μg/ml)

CRP 6.6±0.1 6.0±0.2 0.03 8 6.8±0.1 6.4±0.3 0.14 8

Fibrinogen 6.3±11.0 5.5±0.6 0.18 8 6.5±0.4 6.9±0.3 0.20 7

t-PA (total antigen) <7.3±3.0 nd - 8 nd nd - 7-8

t-PA (active) nd nd - 8 nd nd - 8

__

nd = below threshold of detection

P-values shown are comparison of saline versus DEP, unpaired t-tests.

ApoE^-/-^ were fed Western diet for 8 weeks, whereas wild-type mice were fed normal chow.

**Figure S5**


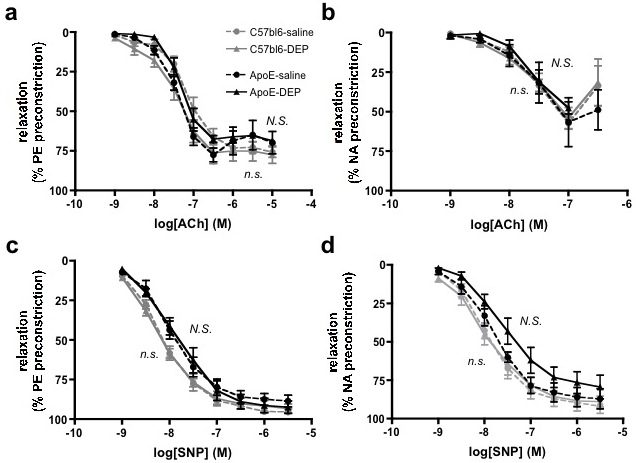


**Figure S5.** Choice of contractile agent does not influence the vasodilator responses in aortic rings from instilled mice. Effect of DEP instillation on **(a, b)** endothelium-dependent relaxation to acetylcholine and **(c, d)** endothelium-independent relaxation to sodium nitroprusside, when vessels are precontracted with either phenylephrine (PE; left panels, a,c) or noradrenaline (NA; right panels, b,d). Response in C57bl6 mice (grey lines) or ApoE^-/-^ mice (black lines) receiving either saline (circles, broken line) or DEP (triangles, solid line) instillation. All dose response curves were performed in the same vessel from a single animal. ApoE^-/-^ were fed Western diet for 8 weeks, whereas wild-type mice were fed normal chow. Mean±S.E.M. (n=4-5 for PE, n=3-7 for NA), ^n.s.^*P*>0.05, two-way ANOVA saline vs DEP in C57bl6 mice, ^N.S.^*P*>0.05, two-way ANOVA saline vs DEP in ApoE^-/-^ mice.

**Supplement References**

1. Miller MR, Borthwick SJ, Shaw CA, McLean SG, McClure D, Mills NL, Duffin R, Donaldson K, Megson IL, Hadoke PW, Newby DE: **Direct impairment of vascular function by diesel exhaust particulate through reduced bioavailability of endothelium-derived nitric oxide induced by superoxide free radicals.** *Environ Health Perspect* 2009, **117:**611-616.

2. Shaw CA, Robertson S, Miller MR, Duffin R, Tabor CM, Donaldson K, Newby DE, Hadoke PW: **Diesel exhaust particulate--exposed macrophages cause marked endothelial cell activation.** *Am J Respir Cell Mol Biol* 2011, **44:**840-851.

3. Rossi AG, Sawatzky DA, Walker A, Ward C, Sheldrake TA, Riley NA, Caldicott A, Martinez-Losa M, Walker TR, Duffin R, et al: **Cyclin-dependent kinase inhibitors enhance the resolution of inflammation by promoting inflammatory cell apoptosis.** *Nat Med* 2006, **12:**1056-1064.

4. Johnson J, Carson K, Williams H, Karanam S, Newby A, Angelini G, George S, Jackson C: **Plaque rupture after short periods of fat feeding in the apolipoprotein E-knockout mouse: model characterization and effects of pravastatin treatment.** *Circulation* 2005, **111:**1422-1430.

5. Cassee FR, Campbell A, Boere AJ, McLean SG, Duffin R, Krystek P, Gosens I, Miller MR: **The biological effects of subacute inhalation of diesel exhaust following addition of cerium oxide nanoparticles in atherosclerosis-prone mice.** *Environ Res* 2012, **115:**1-10.

6. Hadoke P, Wainwright CL, Wadsworth RM, Butler K, Giddings MJ: **Characterization of the morphological and functional alterations in rabbit subclavian artery subjected to balloon angioplasty.** *Coron Artery Dis* 1995, **6:**403-415.

7. Lendrum AC, Fraser DS, Slidders W, Henderson R: **Studies on the character and staining of fibrin.** *J Clin Pathol* 1962, **15:**401-413.

8. Masson P: **Some histological methods: Trichrome staining and their preliminary technique.** *J Technic Meth Bull Int Assoc Med* 1929, **12:**75.

9. Canbay A, Guicciardi ME, Higuchi H, Feldstein A, Bronk SF, Rydzewski R, Taniai M, Gores GJ: **Cathepsin B inactivation attenuates hepatic injury and fibrosis during cholestasis.** *J Clin Invest* 2003, **112:**152-159.

10. Shiomi M, Yamada S, Amano Y, Nishimoto T, Ito T: **Lapaquistat acetate, a squalene synthase inhibitor, changes macrophage/lipid-rich coronary plaques of hypercholesterolaemic rabbits into fibrous lesions.** *Br J Pharmacol* 2008, **154:**949-957.

11. Naghavi M, Libby P, Falk E, Casscells SW, Litovsky S, Rumberger J, Badimon JJ, Stefanadis C, Moreno P, Pasterkamp G, et al: **From vulnerable plaque to vulnerable patient: a call for new definitions and risk assessment strategies: Part II.** *Circulation* 2003, **108:**1772-1778.

12. Naghavi M, Libby P, Falk E, Casscells SW, Litovsky S, Rumberger J, Badimon JJ, Stefanadis C, Moreno P, Pasterkamp G, et al: **From vulnerable plaque to vulnerable patient: a call for new definitions and risk assessment strategies: Part I.** *Circulation* 2003, **108:**1664-1672.

13. Suzuki H, Kobayashi H, Sato F, Yonemitsu Y, Nakashima Y, Sueishi K: **Plaque-stabilizing effect of pitavastatin in Watanabe heritable hyperlipidemic (WHHL) rabbits.** *J Atheroscler Thromb* 2003, **10:**109-116.

14. Rosenfeld ME, Polinsky P, Virmani R, Kauser K, Rubanyi G, Schwartz SM: **Advanced atherosclerotic lesions in the innominate artery of the ApoE knockout mouse.** *Arterioscler Thromb Vasc Biol* 2000, **20:**2587-2592.

15. Jackson CL, Bennett MR, Biessen EA, Johnson JL, Krams R: **Assessment of unstable atherosclerosis in mice.** *Arterioscler Thromb Vasc Biol* 2007, **27:**714-720.

16. Johnson JL, Jackson CL: **Atherosclerotic plaque rupture in the apolipoprotein E knockout mouse.** *Atherosclerosis* 2001, **154:**399-406.

17. Araujo JA, Barajas B, Kleinman M, Wang X, Bennett BJ, Gong KW, Navab M, Harkema J, Sioutas C, Lusis AJ, Nel AE: **Ambient particulate pollutants in the ultrafine range promote early atherosclerosis and systemic oxidative stress.** *Circ Res* 2008, **102:**589-596.

18. Back M, Ketelhuth DF, Agewall S: **Matrix metalloproteinases in atherothrombosis.** *Prog Cardiovasc Dis* 2010, **52:**410-428.

19. Lim CS, Shalhoub J, Gohel MS, Shepherd AC, Davies AH: **Matrix metalloproteinases in vascular disease--a potential therapeutic target?** *Curr Vasc Pharmacol*, **8:**75-85.

20. Reinhart WH: **Fibrinogen--marker or mediator of vascular disease?** *Vasc Med* 2003, **8:**211-216.

21. Miller MR, Grant S, Wadsworth RM: **Selective arterial dilatation by glyceryl trinitrate is not associated with nitric oxide formation in vitro.** *J Vasc Res* 2008, **45:**375-385.
